# Decreased IL-33 in the brain following repetitive mild traumatic brain injury contributes to cognitive impairment by inhibiting microglial phagocytosis

**DOI:** 10.1186/s40779-025-00631-1

**Published:** 2025-08-05

**Authors:** Ze-Xi Jia, Meng-Tian Guo, Mei-Mei Li, Pan Liao, Bo Yan, Wei Zhang, Fang-Yuan Cheng, Ya-Ru Liu, Zi-Han Zhang, Cheng Wei, Jie Zhou, Fang-Lian Chen, Ping Lei, Xin-Tong Ge

**Affiliations:** 1 Department of Geriatrics, Tianjin Medical University General Hospital, Tianjin, 300052 China; 2 Tianjin Geriatrics Institute, Tianjin Medical University General Hospital, Tianjin, 300052 China; 3 Tianjin Neurological Institute, Tianjin Medical University General Hospital, Tianjin, 300052 China; 4 Department of Internal Medicine, Beijing Chao-Yang Hospital, Capital Medical University, Beijing, 100054 China; 5 School of Medicine, Nankai University, Tianjin, 300071 China; 6 Department of Oncology-Pathology, Karolinska Institute, 10339 Stockholm, Sweden; 7 Department of Immunology, School of Basic Medical Sciences, Tianjin Medical University, Tianjin, 300070 China

**Keywords:** Repetitive mild traumatic brain injury (rmTBI), Interleukin-33 (IL-33), Microglia, Cognition

## Abstract

**Background:**

Repetitive mild traumatic brain injury (rmTBI) is a significant risk factor for neurodegeneration, characterized by pathological protein deposition and persistent neuroinflammation. Research has observed increased interleukin-33 (IL-33) levels in the peripheral blood of patients with rmTBI, suggesting IL-33 may participate in regulating the pathological development of rmTBI. The study aims to elucidate the impact and mechanism of IL-33 in the progression of neuropathology following rmTBI, and to explore its potential as a therapeutic target to improve the neurological outcome.

**Methods:**

The study employed an rmTBI mouse model using the wild-type (WT) and *IL-33* knockout mice. Cognitive function was assessed via the Y-maze and Barnes tests. The main cell type expressing IL-33 and its receptor, suppression of tumorigenicity 2 (ST2), was then investigated in the mouse brain through immunofluorescence colocalization. As the primary neural cell responsible for ST2 expression, microglia were studied in vitro using the BV2 cell line. The effects of lipid droplets (LDs) accumulation and amyloid-beta (Aβ) phagocytosis were measured to elucidate the impact of IL-33 on BV2 cells’ phagocytosis. Additionally, HT22 neuronal apoptosis was assessed by flow cytometry. Finally, the cognitive effects of intranasal administration of IL-33 were evaluated in mice.

**Results:**

IL-33KO mice exhibited pronounced cognitive impairment after rmTBI. In the mouse brain, astrocytes were identified as the primary source of IL-33 secretion, while microglia predominantly expressed ST2. Transcriptome sequencing revealed that IL-33 significantly influenced phagocytosis function. IL-33 mitigated LDs accumulation in BV2 cells and enhanced Aβ phagocytosis in vitro. In addition, the culture medium of BV2 cells with activated IL-33/ST2 signaling reduced HT22 neuronal apoptosis and axonal damage. Furthermore, intranasal administration of IL-33 was observed to be effective in alleviating neurodegeneration and cognitive outcome of rmTBI mice.

**Conclusions:**

Dysfunction of the IL-33/ST2 axis following rmTBI leads to cognitive dysfunction via impairing microglial phagocytosis capacity and promoting neuronal damage. IL-33 would be a promising therapeutic target for alleviating neurodegeneration following rmTBI.

**Supplementary Information:**

The online version contains supplementary material available at 10.1186/s40779-025-00631-1.

## Background

Traumatic brain injury (TBI) is an escalating global health issue affecting more than 50 million people annually [1]. It is estimated that the global population will experience at least one TBI in their lifetime [2, 3]. TBI can trigger complex pathological responses in the brain, including axonal injury, neuronal death, glial cell proliferation, blood–brain barrier disruption, and neuroinflammation [4, 5]. It is classified as mild, moderate, or severe based on the injury’s severity and clinical symptoms. Recently, repetitive mild TBI (rmTBI) has garnered increasing attention from neurosurgeons and neurologists. It is considered a significant risk factor of chronic traumatic encephalopathy (CTE), a neurodegenerative disease characterized by abnormal amyloid-beta (Aβ) protein deposition, as well as impaired learning, memory, and cognition [6]. As a newly recognized neurodegenerative disorder, CTE is commonly observed in elderly individuals with declined mobility, military veterans, athletes such as boxers and football players, and victims of domestic violence [7].

Diagnosis of CTE primarily relies on postmortem biopsies conducted according to recognized neuropathological criteria [8]. However, this approach does not facilitate the timely diagnosis or treatment of affected patients. Therefore, identifying effective diagnostic biomarkers and investigating therapeutic targets to mitigate neuro-pathological progression are crucial. In our preliminary study, it was found that interleukin-33 (IL-33) could serve as a promising serum and exosomal biomarker for rmTBI, based on an established clinical cohort [9]. IL-33 is a key member of the IL-1 cytokine family and plays an important role in various diseases. Its impact is largely mediated through its interactions with suppression of tumorigenicity 2 (ST2), a receptor that is vital for many immune responses, and the accessory receptor interleukin-1 receptor accessory protein (IL-1RAcP) [10–12]. In the central nervous system (CNS), astrocytes are the primary cells expressing IL-33, while the expression levels in microglia, oligodendrocytes, and neurons are relatively low [13, 14]. Under normal physiological conditions, IL-33 is mainly localized in the nucleus. However, when cells are damaged or exposed to inflammatory stimuli, IL-33 may be passively released into the extracellular environment, thereby triggering an immune response [15, 16]. Furthermore, ST2 was initially identified in mouse fibroblasts, but it has also been observed in microglia and astrocytes later on. The ST2 protein mainly exists in 2 forms. One type of receptor is the membrane-bound form of ST2, which is present on the surface of various immune cells that can bind to IL-33. Additionally, there is a soluble variant known as soluble ST2, which acts as a decoy receptor for IL-33, capturing free IL-33 and thus modulating the associated signaling pathways [15, 17]. Recent evidence has highlighted the crucial role of the IL-33/ST2 pathway in Alzheimer’s disease (AD), Parkinson’s disease (PD), and several other neurological conditions [10, 18, 19]. Phi T. Nguyen’s research revealed that IL-33-mediated neuron-microglia communication promotes dendrite formation, enhances synaptic plasticity, and is critical for learning and memory capacity [20]. Moreover, recent studies suggested that IL-33 administration has the potential to counteract deficits in synaptic plasticity and improve learning, along with memory impairments in amyloid precursor protein (APP)/presenilin 1 mouse models [13, 18, 20]. In the middle cerebral artery occlusion model, IL-33 treatment has been shown to reduce the infarct area and improve the neurological outcomes [21]. Despite these findings, the role of IL-33 in the pathological progression following rmTBI remains unclear.

This study aims to clarify the impact of IL-33 on cognitive outcomes related to rmTBI and the underlying mechanisms involved. Furthermore, we designed a therapeutic strategy targeting the IL-33/ST2 axis to alleviate cognitive impairment in rmTBI mice. The findings may provide valuable insights that could inform clinical strategies for the diagnosis and treatment of rmTBI.

## Methods

### Human blood samples

The patient study was conducted as part of the clinical trial titled Observational Cohort Study of Blood Transcriptomics and Proteomics Information as Biomarkers of Traumatic Encephalopathy Syndrome (ClinicalTrials.gov No. NCT04928534), in accordance with the Declaration of Helsinki. Approval for the inclusion of human participants was granted by the Ethics Committee of Tianjin Medical University General Hospital (IRB2021-YX-056–01).

From August 2021 to July 2022, blood samples were collected from 6 patients with rmTBI at the Department of Neurosurgery of Tianjin Medical University General Hospital. Additionally, blood samples were collected from 6 sex- and age-matched healthy individuals. The blood exosomes were extracted and characterized according to the methods described in Additional file 1: Methods. The inclusion criteria for patients were as follows: 1) age between 18 and 80 years, with the ability to perform daily activities independently; 2) a confirmed history of rmTBI; and 3) the latest head injury occurring no later than 3 months prior to participation. Exclusion criteria included comorbidities that could confound results, such as pregnancy, previous moderate-to-severe TBI, severe systemic diseases, and neuropsychiatric conditions.

### Animals and treatments

Adult male C57BL/6J mice (*n* = 120) aged 8 to 10 weeks were sourced from the Beijing Vital River Laboratory Animal Technology Co., Ltd. (Beijing, China), and *IL-33* CRISPR knockout (IL-33^−/−^) mice (*n* = 80) were obtained from the Shanghai Model Organisms Center, Inc. (Shanghai, China). The animals were housed in a standard animal care facility, provided with rodent chow and sterile water, and kept on a 12 h light/dark cycle. All procedures were approved by the Animal Care and Use Committee at Tianjin Medical University (IRB2024-DWFL-044) and complied with the National Institutes of Health Guidelines for the Care and Use of Laboratory Animals. In order to minimize the potential impact of sex differences on experimental results, all the experimental mice used in our study were male. Following rmTBI, mice were randomly assigned to receive either intranasal recombinant IL-33 protein (MCE, USA; 2 µg/30 g body weight) or a vehicle control at specific intervals (days 0, 7, 14, 21, 28, 35, and 42) [22–24]. Our experiment used a total of 200 mice, divided into 6 groups: WT-Sham (*n* = 40), WT-rmTBI (*n* = 40), IL-33KO-Sham (*n* = 40), IL-33KO-rmTBI (*n* = 40), rmTBI + phosphate buffer solution (PBS) (*n* = 20), and rmTBI + IL-33 (*n* = 20).

### Mouse model

The rmTBI mouse model was developed following previously established methodologies [25]. The fur on the mice’s heads was shaved, and anesthesia was induced using 4.6% isoflurane. The mice were then placed prone on an acrylic mold and secured with surgical tape across their shoulders, leaving a 3.0 mm space beneath the head to allow for acceleration and deceleration during impact. A custom-made concave metal disc (3.0 mm in diameter) was affixed to the posterior skull near the anterior fontanelle, functioning as a helmet to transmit the force across the brain and induce mild diffuse brain injury, mimicking clinical rmTBI pathology. Using a controlled cortical impact (CCI) device, the impact was applied at the center of the parietal bone at a velocity of 5.0 m/s, achieving a mouse head displacement of 2.5 mm. This procedure was repeated 4 times, with 48 h intervals between impacts, to model repetitive mild injury. Sham-operated mice underwent the same procedures, except for the impact. Behavioral performance was evaluated using the Barnes maze and Y-maze tests. The details of the experiments are shown in Additional file 1: Methods.

### Cell culture

BV2 microglial cells and HT22 neuronal cells were purchased from Wuhan Procell Life Science & Technology Co., Ltd. The cells used in this study were cultured in Dulbecco’s modified Eagle medium (DMEM, Gibco, USA), which was enriched with a 1% solution of penicillin and streptomycin to ensure the prevention of bacterial contamination. The medium also contained 10% fetal bovine serum, providing essential nutrients and growth factors necessary for optimal cell growth and maintenance. Both cell lines were incubated in a humidified environment at a temperature of 37 °C, with a consistent concentration of 5% CO_2_, which is crucial for sustaining the physiological conditions required for cellular proliferation and viability. Aβ phagocytosis assay and apoptosis assay were conducted according to the methods described in Additional file 1: Methods.

### Cell transfection

siRNA dry powder was dissolved in diethyl pyrocarbonate-treated water according to the instructions. A total of siRNA (5 µl, sense strand sequence: GGUAUUACUCAGAUACAAATT; antisense strand sequence: UUUGUAUCUGAGUAAUACCTT) and Lipofectamine RNAiMAX reagent (5 µl, 13778–075, Invitrogen, USA) were mixed in DMEM medium (150 µl) and incubated for 5 min. The mixture was added to DMEM medium containing 10% fetal bovine serum and incubated in the incubator for 24 − 48 h.

### Western blotting

Western blotting was performed according to our established protocol [26]. Briefly, tissue samples were lysed using a buffer containing high-efficiency RIPA lysis buffer, phosphatase inhibitors, and protease inhibitors to extract proteins. Proteins were separated using 12.5% SDS–polyacrylamide gels for Alix (1:1000, 2171S, Cell Signaling Technology, USA), CD9 (1:1000, 13403S, Cell Signaling Technology, USA), CD63 (1:1000, ab134045, Abcam, UK), APP (1:1000, 2452S, Cell Signaling Technology, USA), tau (1:1000, 4019S, Cell Signaling Technology, USA), phospho-tau (p-tau; 1:1000, 49561S, Cell Signaling Technology, USA), β-actin (1:1000, 4970S, Cell Signaling Technology, USA), and ST2 (1:1000, PA5-20077, Invitrogen, USA). Protein bands were analyzed with the ChemiDoc XRS imaging system (Bio-Rad, USA), and band intensities were quantified using ImageJ software to determine relative density.

### Immunofluorescence staining

After deep anesthesia, mice underwent cardiac perfusion with PBS and 4% paraformaldehyde. After fixation, tissues were dehydrated using a sucrose gradient solution until fully submerged. Samples were cryo-sectioned after embedding in optimal cutting temperature. The sections underwent immunofluorescence staining.

Tissues were warmed to room temperature for 20 min in a humidified chamber. The sections were washed 3 times with PBS to remove the optimal cutting temperature. Excess moisture was removed from the sections, which were then blocked for 1.5 h with blocking buffer. Primary antibodies were applied and incubated overnight at 4 °C. The primary antibodies used included IL-33 (1:200, AF3626, R&D, USA), CD31 (1:200, A19014, Abclonal, China), oligodendrocyte lineage transcription factor 2 (Olig2, 1:200, OB-PGP040, Oasis Biofarm, China), glial fibrillary acidic protein (GFAP, 1:200, 3670S, Cell Signaling Technology, USA), transmembrane protein 119 (TMEM119, 1:200, 90840S, Cell Signaling Technology, USA), neuron-specific nuclear protein (NeuN, 1:200, NBP1-92693, Novus Biologicals, USA), ionized calcium-binding adapter molecule 1 (Iba1, 1:200, OB-PGP049, Oasis Biofarm, China), ST2 (1:200, PA5-20077, Invitrogen, USA), CD68 (1:200, sc-20060, Santa Cruz Biotechnology, USA), lysosome-associated membrane protein 1 (LAMP1, 1:200, sc-20011, Santa Cruz Biotechnology, USA), and APP (1:200, AF7687, Affinity Biosciences, USA).

On the next day, the sections were rewarmed from the refrigerator for 20 min, washed to remove the primary antibody, and incubated with the secondary antibody for 1 h under light protection. Sections were observed under a microscope (Olympus, Japan). Details of cell immunofluorescence staining and Sholl analysis of microglial morphology are shown in Additional file 1: Methods.

### Microtubule-associated protein tau metabolism determined by ^18^F‑S16‑tau using positron emission tomography/computed tomography (PET/CT)

After anesthetizing the mice, each mouse was injected via the tail vein with 5 MBq of ^18^F-fluoro-2-deoxy-D-glucose. After 30 min, the mice were anesthetized with 2% isoflurane gas and placed in the bed of a micro-PET/CT scanner (Novel Medical, Beijing, China). CT scan parameters were as follows: tube voltage 80 kV; tube current 0.5 mA; field of view 70 mm; slice thickness 0.18 mm. The PET scan parameters were as follows: field of view 70 mm; matrix 140 × 140; reconstruction protocol PET-ordered subset expectation maximization (OSEM)-recon; iterations: 40.

### RNA sequencing

RNA sequencing was performed by GenDenovo Biotechnology Co., Ltd. (Guangzhou, China). Total RNA was extracted from mouse brain tissue using TRIzol reagent (Invitrogen, USA). Polyadenylated mRNA was enriched using oligo (dT) magnetic beads, fragmented by ultrasonication, and reverse-transcribed into cDNA. The purified double-stranded cDNA was end-repaired, A-tailed, and adapter-ligated. Fragments of about 200 bp were size-selected using AMPure XP beads. Finally, PCR amplification was performed, and the products were purified with AMPure XP beads to generate the final library. Differentially expressed genes were mapped to the Kyoto Encyclopedia of Genes and Genomes (KEGG) database for pathway enrichment analysis. Gene Set Enrichment Analysis (GSEA) was performed using GSEA software, with significance thresholds set at |NES|> 1, nominal *P*-value (NOM *P*-val) < 0.05, and false discovery rate < 0.25.

### Statistical analysis

Data were presented as means ± standard error of the mean (SEM) and analyzed with GraphPad Prism 9.0. For comparisons across three or more groups, a one-way ANOVA followed by Tukey’s post hoc test was used. Comparisons of two groups utilized the Student’s *t*-test. A *P*-value of less than 0.05 was considered statistically significant.

## Results

### The dynamic change of IL-33 levels following rmTBI

**Fig. 1:**
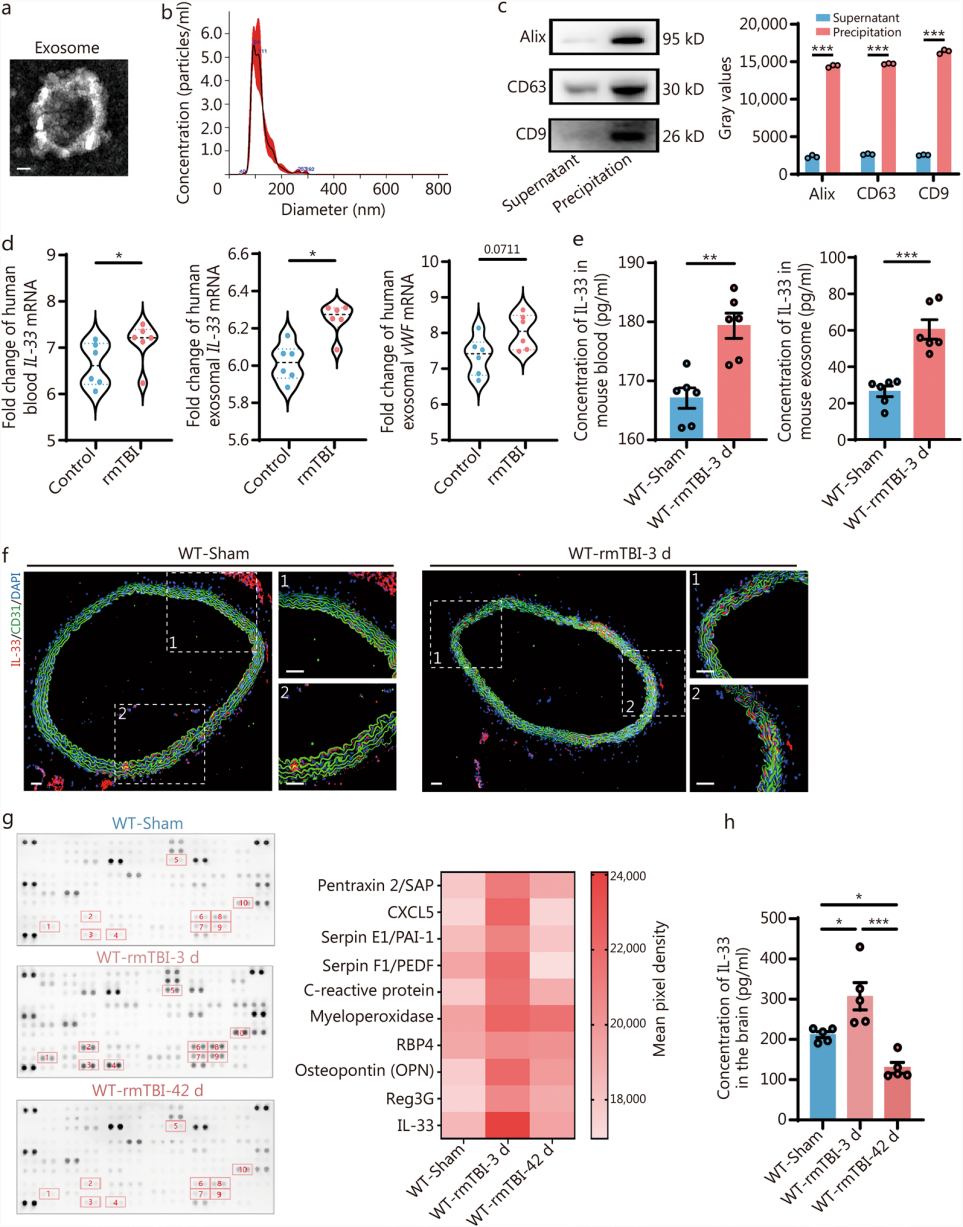
The dynamic change of IL-33 levels following rmTBI.** a** TEM image displaying purified exosomes. Scale bar = 1 μm. **b** Analysis of size distribution for purified exosomes conducted via NTA. **c** Western blotting analysis of characteristic exosomal biomarkers, including Alix, CD63, and CD9. **d** mRNA levels of total *IL-33*, exosomal *IL-33*, and *vWF* in blood from patients with rmTBI and healthy control individuals (*n* = 6). **e** ELISA measurements of total circulating IL-33 and exosomal IL-33 in blood at specified time points post-rmTBI (*n* = 6). **f** Representative immunofluorescence staining images showing IL-33 (red), CD31 (green), and DAPI (blue) in the aortas of mice at specified time points post-rmTBI. Scale bar = 50 μm. **g** Array data were quantified using ImageJ to generate a protein profile, which is displayed in a heatmap. **h** ELISA was performed to measure IL-33 levels in the mouse brain at specified time points post-rmTBI (*n* = 5). Data are represented as the mean ± SEM. ^*^P < 0.05, ^**^P < 0.01, ^***^P < 0.001. TEM transmission electron microscope, NTA nanoparticle tracking analysis, DAPI 4,6-diamidino-2-phenylindole dihydrochloride, rmTBI repetitive mild traumatic brain injury, vWF von Willebrand factor, ELISA enzyme-linked immunosorbent assay

IL-33 plays a crucial role as a cytokine that facilitates intercellular communication. This cytokine is not only significant for local signaling but can also influence distant target cells. To investigate IL-33 activation following rmTBI in peripheral endothelial cells, we collected blood exosomes from rmTBI patients and healthy controls (baseline characteristics) (Additional file 1: Table S1). The morphology and size of these exosomes were assessed using transmission electron microscope (TEM) and nanoparticle tracking analysis (NTA). TEM imaging showed the particles to be round, with diameters ranging from 30 to 150 nm. This size distribution was complemented by NTA results, which showed a peak diameter of 94 nm. Western blotting analysis revealed significant expression of surface exosomal markers, including Alix, CD63, and CD9, in the precipitates. In contrast, these markers were significantly reduced in the supernatants (Fig. 1a-c). IL-33 levels were significantly increased in the blood and exosomes of patients with rmTBI. Furthermore, these IL-33^+^ exosomes showed significant expression of the endothelial marker von Willebrand factor (vWF) (Fig. 1d). This phenomenon was confirmed in a mouse model, where circulating exosomes in the blood were collected from both WT-rmTBI and WT-sham mice, and IL-33 levels were quantified using enzyme-linked immunosorbent assay (ELISA). Results showed a significant increase in IL-33 levels in the blood and exosomes on day 3 post-rmTBI compared to the WT-sham group (Fig. 1e). Immunofluorescence staining further demonstrated increased IL-33 expression in the aortic endothelial cells of WT-rmTBI mice (Fig. 1f). Additionally, we explored the role of IL-33 in the CNS following rmTBI. IL-33 expression was evaluated in both the acute and chronic phases following the injury (Fig. 1g**)**. The ELISA findings showed an increase in IL-33 levels in the mouse brain on the third day after rmTBI, which then decreased by day 42 (Fig. 1h**)**.

### Cellular localization of IL-33 and ST2 in the WT-rmTBI mouse brain

**Fig. 2:**
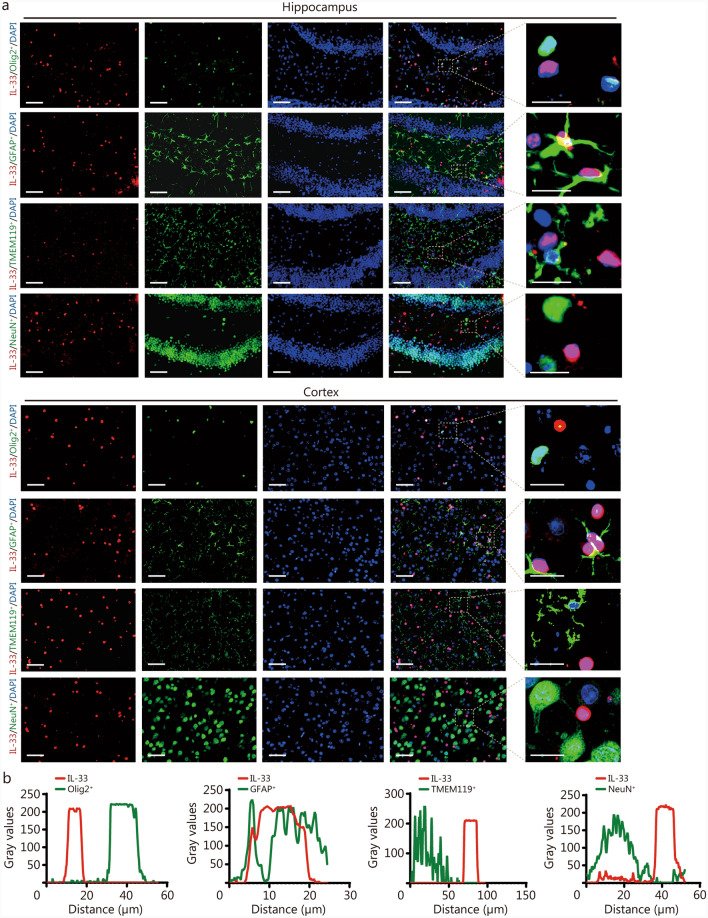
Cellular localization of IL-33 in the hippocampal (dentate gyrus) and cortical regions of WT-rmTBI mice. **a** Representative immunofluorescence staining images of IL-33 (red), Olig2^+^ oligodendrocytes (green), GFAP^+^ astrocytes (green), TMEM119^+^ microglia (green), NeuN^+^ neurons, and DAPI (blue) in the hippocampus and cortex of mice on day 42 post-rmTBI (*n* = 5, 3 slides/mouse. Consistent fields of view were selected across all slides for subsequent quantification). Scale bar = 50 μm (main images) and 25 μm (magnified insets). **b** Fluorescence intensity plots of IL-33 and various cells. The red curves show the relative intensity of IL-33, and the green curves show Olig2^+^ oligodendrocytes, GFAP^+^ astrocytes, TMEM119^+^ microglia, and NeuN^+^ neurons. DAPI 4,6-diamidino-2-phenylindol dihydrochloride, IL-33 interleukin-33, Olig2 oligodendrocyte lineage transcription factor 2, GFAP glial fibrillary acidic protein, TMEM119 transmembrane protein 119, NeuN neuron-specific nuclear protein, WT wild-type, rmTBI repetitive mild traumatic brain injury

Previous studies have indicated that astrocytes in the CNS are the primary sites for IL-33 localization [13, 27, 28], but the cellular distribution of IL-33 and its receptor ST2 after rmTBI remains unclear. In this study, we performed double immunofluorescence staining for IL-33 and ST2 with Olig2 (oligodendrocyte marker), GFAP (astrocyte marker), TMEM119/Iba1 (microglia marker), and NeuN (neuron marker). On day 42 post-rmTBI, strong co-localization of IL-33 and GFAP was observed in the hippocampus and cortex, predominantly in the nuclei, with minimal overlap with Olig2, TMEM119, and NeuN (Fig. 2a). ST2 was primarily co-localized with Iba1 (Additional file 1: Fig. S1a). Fluorescence intensity analysis further confirmed these findings (Fig. 2b; Additional file 1: Fig. S1b). These findings indicate that astrocytes are the main source of IL-33 in the CNS, while ST2 is predominantly expressed in microglia after rmTBI.

### IL-33 deficiency exacerbated cognitive deficits induced by rmTBI

**Fig. 3:**
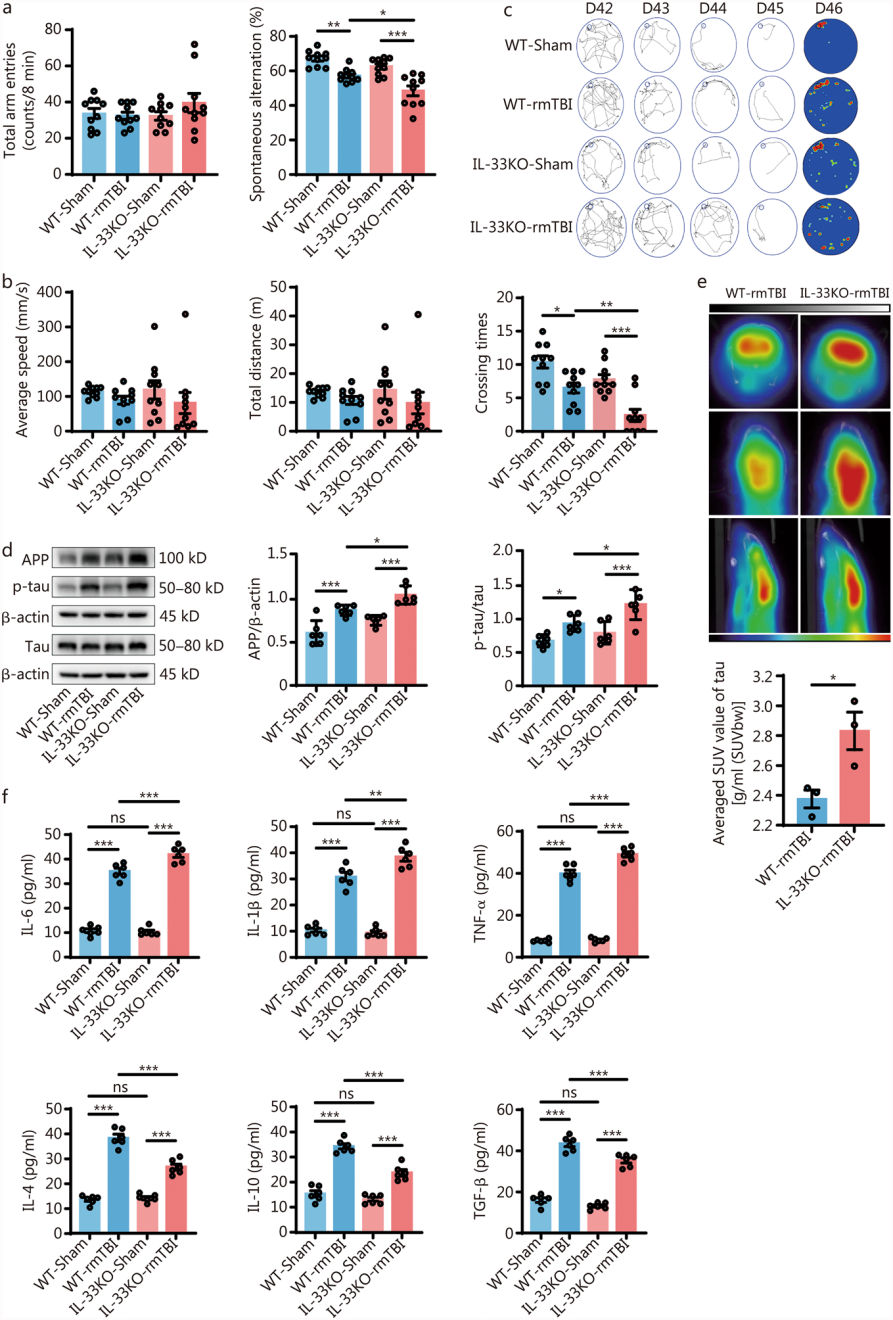
IL-33 deficiency exacerbated cognitive deficits induced by rmTBI. **a** Total number of entries and percentage of spontaneous alternations in the Y-maze (*n* = 10). **b** Average speed, total distance, and crossing times during the exploration phase of the Barnes maze for mice (*n* = 10). **c** Training and spatial exploration paths of mice in the Barnes maze experiment from days 42 to 46 post-rmTBI. **d** Western blotting analysis of APP, p-tau, and tau on day 42 post-rmTBI (*n* = 6). **e** Reconstructed S16-tau PET/CT images of mice and average SUV values in the hippocampal region of the mouse brain (*n* = 3). **f** ELISA measurements of IL-6, IL-1β, TNF-α, IL-4, IL-10, and TGF-β in the mouse brain on day 42 post-rmTBI (*n* = 6). Data are represented as the mean ± SEM. ^*^P < 0.05, ^**^P < 0.01, ^***^P < 0.001, ns non-significant. DAPI 4,6-diamidino-2-phenylindol dihydrochloride, PET/CT positron emission tomography/computed tomography, SUV standardized uptake value, WT wild-type, KO knockout, APP amyloid precursor protein, rmTBI repetitive mild traumatic brain injury, ELISA enzyme-linked immunosorbent assay, IL interleukin, TNF-α tumor necrosis factor-α, TGF-β transforming growth factor-β, SUVbw standardized uptake value based on body weight

As a significant risk factor for the onset and progression of neurodegenerative diseases, rmTBI is characterized by the abnormal accumulation of Aβ and tau proteins, along with cognitive deficits [29]. Previous studies have shown that IL-33 is essential for preserving cognitive function in mice [18, 28, 30]. Thus, we examined the impact of IL-33 deficiency on cognitive performance following rmTBI. Memory and learning abilities were assessed using the Y-maze and Barnes maze tests on day 42 post-injury. In sham-operated IL-33KO mice, there were no significant differences in behavioral performance compared to WT controls. However, following rmTBI, IL-33KO mice showed a reduced percentage of correct alternations in the Y-maze test, despite no changes in the total number of arm entries (Fig. 3a). A similar trend was observed in the Barnes maze test. Although there were no significant differences in average speed or total distance traveled after rmTBI, IL-33KO mice displayed fewer visits to the target hole (Fig. 3b, c). These results suggest that IL-33 deficiency leads to impaired spatial learning and memory skills after rmTBI. We further investigated the impact of IL-33 deficiency on the accumulation of pathological proteins after rmTBI. Western blotting analysis revealed a significant increase in the levels of APP and p-tau/tau in the WT-rmTBI group compared to WT-sham controls. This pathological elevation was even more pronounced in IL-33KO-rmTBI mice (Fig. 3d).

Additionally, small animal PET/CT was used to assess brain imaging changes in mice. Using the [^18^F] S16-tau probe to detect tau deposition, it was found that the standardized uptake value of S16-tau in the hippocampus was significantly higher in IL-33KO-rmTBI mice compared to WT-rmTBI mice (Fig. 3e). IL-33 deficiency also appeared to influence the expression of inflammatory factors in the brain. The results showed that IL-33KO mice had increased levels of pro-inflammatory cytokines, including IL-6, IL-1β, and tumor necrosis factor-α (TNF-α), after rmTBI. In contrast, the levels of anti-inflammatory cytokines, including IL-4, IL-10, and transforming growth factor-β (TGF-β), were lower in IL-33KO mice compared to WT mice (Fig. 3f). Overall, these findings demonstrate that IL-33 deficiency exacerbates cognitive deficits and promotes chronic neuroinflammatory responses following rmTBI.

### IL-33 deficiency impaired microglial phagocytic function

**Fig. 4:**
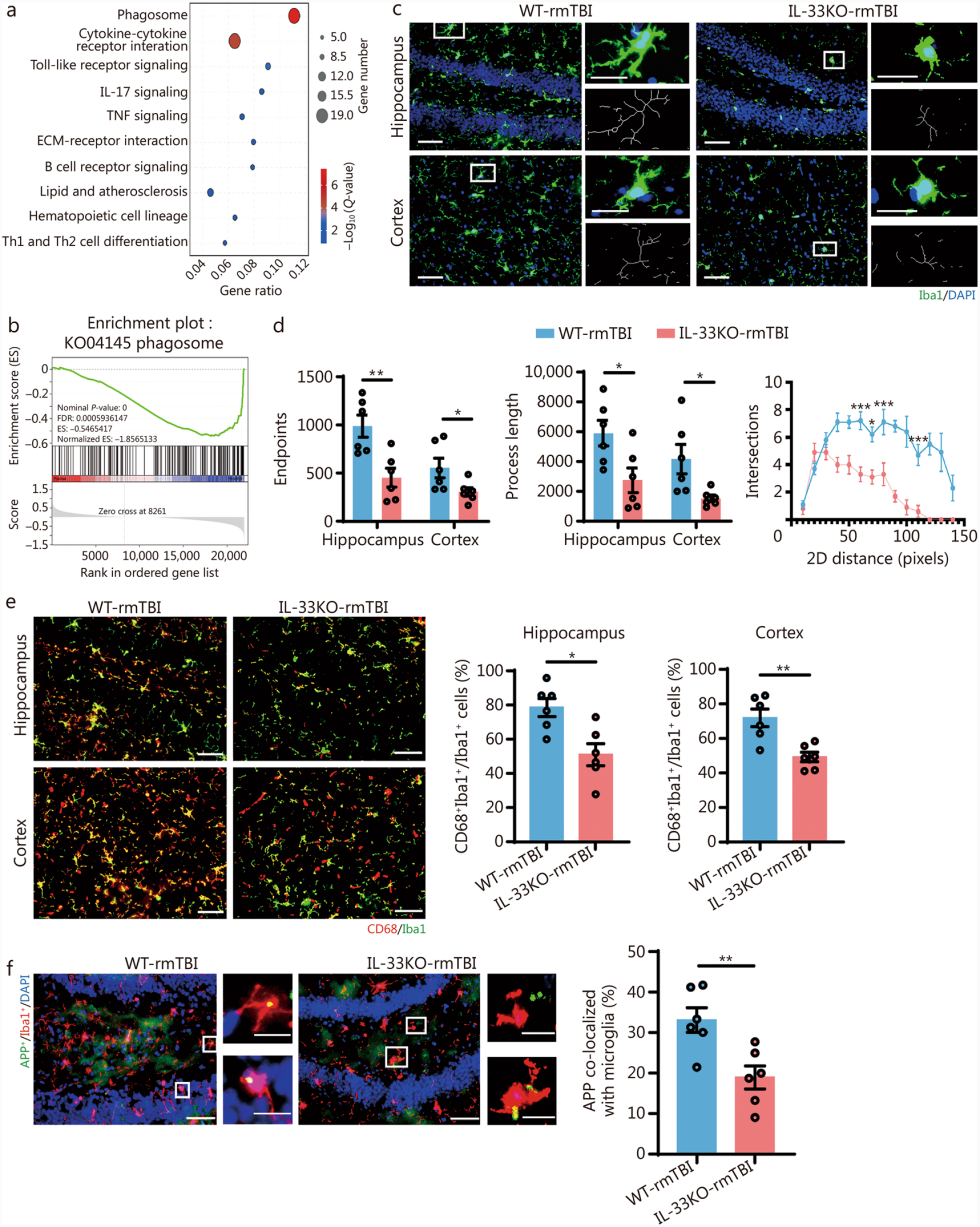
IL-33 deficiency impaired microglial phagocytic function. **a** KEGG enrichment scatter plot showing representative differential pathways between IL-33KO and WT groups at 42 d post-rmTBI. **b** GSEA analysis related to the phagocytic process in IL-33KO-rmTBI mice. **c** Representative immunofluorescence staining images of Iba1^+^ microglia (green) and DAPI (blue) in the hippocampus and cortex of WT-rmTBI and IL-33KO-rmTBI mice. Scale bar = 50 μm (main images) and 20 μm (magnified insets). **d** Endpoint counts, process length, and Sholl analysis of Iba1^+^ cells (*n* = 6). **e** Representative immunofluorescence staining images and quantification of CD68^+^ (red), Iba1^+^ microglia (green) in the hippocampus and cortex of WT-rmTBI and IL-33KO-rmTBI mice (*n* = 6). Scale bar = 50 μm. **f** Representative immunofluorescence staining images and quantification of APP^+^ (green), Iba1^+^ microglia (red), and DAPI (blue) in the hippocampus of WT-rmTBI and IL-33KO-rmTBI mice (*n* = 6). Scale bar = 50 μm (main images) and 20 μm (magnified insets). Data are represented as the mean ± SEM. ^*^P < 0.05, ^**^P < 0.01, ^***^P < 0.001. DAPI 4,6-diamidino-2-phenylindol dihydrochloride, KEGG Kyoto Encyclopedia of Genes and Genomes, GSEA Gene Set Enrichment Analysis, rmTBI repetitive mild traumatic brain injury, Iba1 ionized calcium-binding adapter molecule 1, APP amyloid precursor protein, IL-33 interleukin-33, TNF tumor necrosis factor, ECM extracellular matrix, Th T helper cell, WT wild-type, KO knockout

To further investigate the potential mechanisms by which IL-33 influences cognitive function in mice with rmTBI, we performed transcriptomic analysis on brain tissue (bilateral cerebral cortex and hippocampus) from both WT and IL-33KO groups following rmTBI. These regions were chosen as the primary sites of APP deposition and abnormal phosphorylation of tau proteins in the rmTBI model. Additionally, the hippocampus is mainly responsible for learning, memory, and cognitive functions. KEGG pathway analysis and GSEA revealed a notable increase in biological processes linked to phagocytic activity (Fig. 4a, b). At the same time, we validated the level changes of different expression genes found by transcriptomic analysis. Among them, the key gene *Fcgr4* was selected, which enhances microglial phagocytosis by binding to IgG in immune complexes [31, 32]. It is particularly important in the clearance of cellular debris and pathogens. The results of real-time quantitative polymerase chain reaction (RT-qPCR) showed reduced expression of *Fcgr4* in the brain of mice in the IL-33KO group after rmTBI compared to the WT group (Additional file 1: Fig. S2a, b). Both our findings and previous studies have shown that the ST2 receptor is primarily expressed in microglia within the CNS [19, 33–35]. Therefore, we investigated the impact of IL-33 on microglial phagocytic function. Using ImageJ software, we analyzed the morphology of microglia. The results revealed that after rmTBI, IL-33KO mice exhibited a significant reduction in the number of endpoint terminals and shorter process lengths in both the hippocampal and cortical regions compared to the WT group. Additionally, Sholl analysis showed a marked decrease in the number of intersections at various radii in IL-33KO mice (Fig. 4c, d). We performed additional experiments to compare microglial morphology in the IL-33KO mice and the WT mice without rmTBI. Immunofluorescence staining for Iba1 was performed to observe the morphological changes of microglia. We noted that there was no significant difference between the 2 groups (Additional file 1: Fig. S3a).

CD68, a transmembrane glycoprotein predominantly expressed in intracellular lysosomes of monocytes and macrophages, serves as a scavenger receptor for debris clearance [36]. The intensity of CD68 fluorescence labeling lysosomes was used as a measure, revealing reduced lysosomal content in the hippocampal and cortical microglia of IL-33KO-rmTBI mice, indicating impaired phagocytic ability (Fig. 4e). We compared the expression patterns of LAMP1 and CD68 to ensure the accuracy of lysosomal labelling (Additional file 1: Fig. S3b).

Abnormal processing of APP membrane proteins produces Aβ amyloid plaques, causing nerve damage and cognitive decline. rmTBI promotes abnormal APP processing and disrupts the blood–brain barrier, allowing harmful substances to enter the brain, which may further interfere with APP metabolism [37–39]. For instance, previous research has shown that IL-33 in the brain reduces soluble Aβ levels and amyloid plaque deposition in a mouse model of AD [18]. Given that microglial phagocytic activity directly affects the clearance of APP [40], we assessed the phagocytic capacity of resident hippocampal microglia for APP following IL-33 deficiency. To mediate APP phagocytosis and uptake, microglia must first be recruited to the vicinity of APP and then activated to engulf it. Results showed that microglia were actively phagocytosing APP in the brains of WT-rmTBI mice, whereas IL-33 deficiency hindered the recruitment and phagocytosis of APP by microglia (Fig. 4f; Additional file 1: Fig. S3c). These findings suggest that IL-33 deficiency impairs microglial phagocytic function, leading to increased APP deposition.

### IL-33 improved phagocytic function by reducing lipid droplets (LDs) accumulation in BV2 microglial cells

**Fig. 5:**
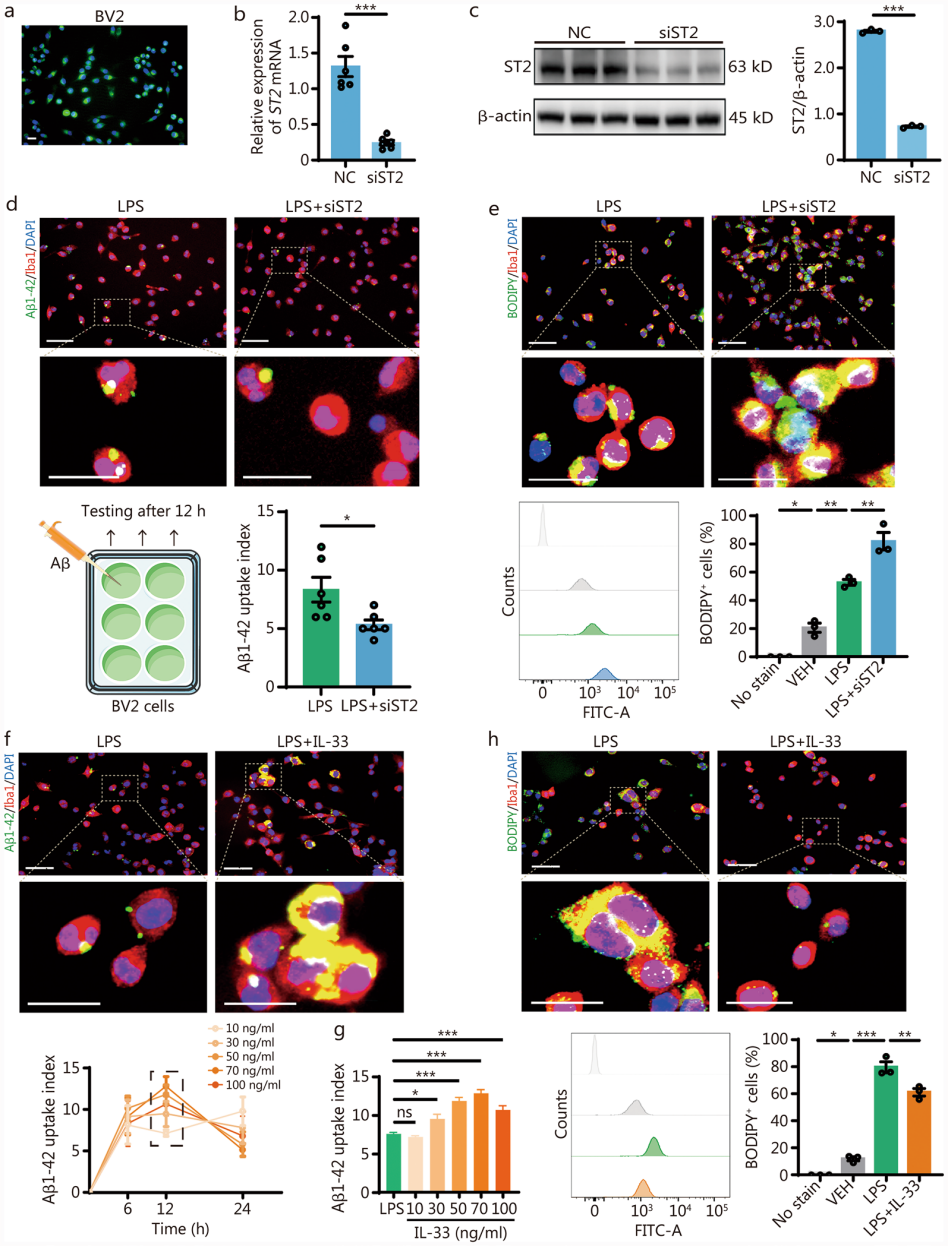
IL-33 improved phagocytic function by reducing LD accumulation in BV2 microglial cells. **a** Representative image of siRNA-ST2 (green) transfected BV2 cells. Scale bar = 50 µm. RT-qPCR (**b**) and Western blotting (**c**) analysis showing changes in ST2 levels in BV2 cells following transfection. **d** Representative immunofluorescence image of Iba1^+^ BV2 cells (red) after 12 h of treatment with Aβ1-42 (green) and uptake index of Aβ1-42 in BV2 cells. Scale bar = 50 μm (main images) and 25 μm (magnified insets). **e** Representative immunofluorescence staining images of BODIPY^+^ (green) in BV2 cells. Scale bar = 50 μm (main images) and 25 μm (magnified insets). Quantification was performed by flow cytometry (*n* = 3). **f** Representative immunofluorescence image of Iba1^+^ BV2 cells (red) after 12 h of treatment with Aβ1-42 (green) following IL-33 intervention. Scale bar = 50 μm (main images) and 25 μm (magnified insets). **g** The uptake of Aβ1-42 by BV2 cells under different concentrations of IL-33 intervention (*n* = 6). **h** Representative immunofluorescence staining images of BODIPY^+^ (green) in BV2 cells after IL-33 intervention. Scale bar = 50 μm (main images) and 25 μm (magnified insets). Quantification was performed by flow cytometry (*n* = 3). Data are represented as the mean ± SEM. ^*^P < 0.05, ^**^P < 0.01, ^***^P < 0.001, ns non-significant. DAPI 4,6-diamidino-2-phenylindol dihydrochloride, ST2 suppression of tumorigenicity 2, LPS lipopolysaccharide, Aβ1-42 amyloid-beta 1–42, Iba1 ionized calcium-binding adapter molecule 1, BODIPY boron-dipyrromethene, VEH vehicle, IL-33 interleukin-33, FITC fluorescein isothiocyanate, LDs lipid droplets, RT-qPCR real-time quantitative polymerase chain reaction

To investigate in more depth the precise mechanism through which IL-33 influences the phagocytic activity of microglia, BV2 cells were cultured in vitro. Initially, we transfected ST2 into BV2 cells, and the transfection efficiency was confirmed using RT-qPCR and Western blotting (Fig. 5a-c). To further investigate the impact of IL-33 on microglial phagocytosis, we performed uptake assays using fluorescein isothiocyanate (FITC)-labeled Aβ after stimulating BV2 microglial cells with lipopolysaccharide (LPS). The results showed that *ST2* knockdown reduced both the uptake capability and efficiency of FITC-Aβ in BV2 cells (Fig. 5d). In neurodegenerative diseases such as AD, the accumulation of LDs in microglia impairs phagocytic function and exacerbates pathological protein deposition [41, 42]. Interestingly, LPS treatment of BV2 cells induced LD formation, and *ST2* knockdown further exacerbated LD accumulation (Fig. 5e).

Subsequently, we tested whether treating BV2 cells with IL-33 could improve this phenomenon. To determine the optimal timing and concentration for IL-33 intervention, BV2 cells were exposed to various conditions. After 12 h of LPS treatment, the highest uptake of FITC-Aβ was observed in BV2 cells (Fig. 5f). Following that, we exposed BV2 cells to different concentrations of IL-33, determining that 50 ng/ml was the most effective concentration (Fig. 5g). In this system, we observed that IL-33 treatment restored the ability of BV2 cells to uptake Aβ while simultaneously reducing LD accumulation (Fig. 5h). These findings indicate that inhibiting the IL-33/ST2 pathway in BV2 cells may exacerbate cognitive impairment in part by impairing Aβ clearance, with LD accumulation potentially contributing to this effect.

### IL-33/ST2 signaling activation in BV2 cells contributed to their neuroprotective effects in vitro

**Fig. 6:**
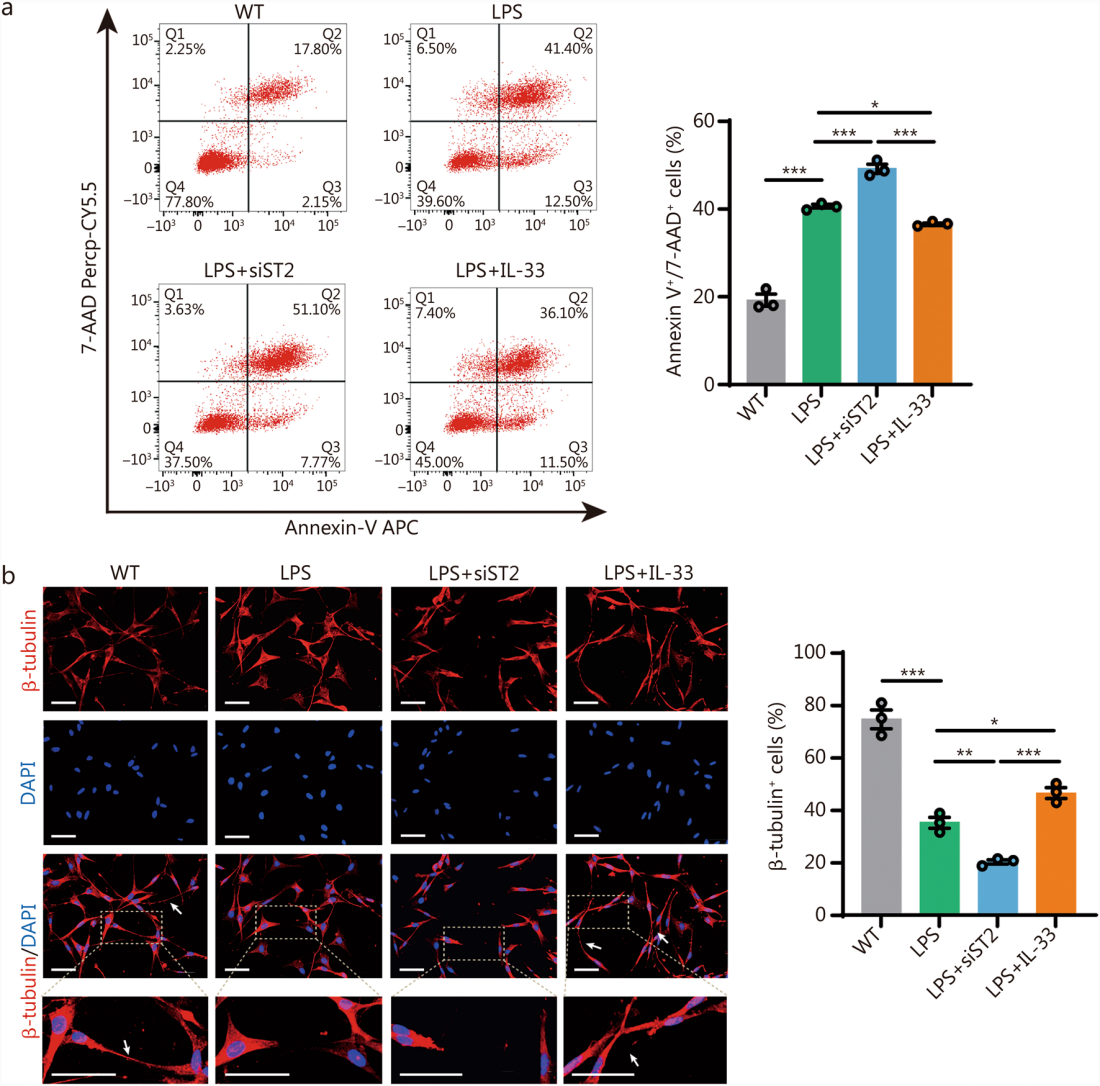
IL-33/ST2 signaling activation in BV2 cells contributed to their neuroprotective effects in vitro. **a** Flow cytometric analysis of apoptosis after different treatments (*n* = 3). **b** Representative immunofluorescence staining images and quantification of β-tubulin (red) and DAPI (blue) following different treatments (*n* = 3). Scale bar = 50 μm (main images) and 25 μm (magnified insets). Arrows indicate axons. Data are represented as the mean ± SEM. ^*^P < 0.05, ^**^P < 0.01, ^***^P < 0.001. DAPI 4,6-diamidino-2-phenylindol dihydrochloride, WT wild-type, LPS lipopolysaccharide, ST2 suppression of tumorigenicity 2, IL-33 interleukin-33, 7-AAD 7-aminoactinomycin D, APC allophycocyanin

It is already known that ST2 is almost undetectable in neurons following rmTBI. To analyze the effects of the IL-33/ST2 axis in microglia on neurons, we treated BV2 cells with 50 ng/ml IL-33 for 12 h. The conditioned media from these BV2 cells were then collected and used to treat HT22 neurons for 24 h. Flow cytometry results showed that conditioned media from *ST2* knockdown BV2 cells increased HT22 neuronal apoptosis, while conditioned media from IL-33-treated BV2 cells reduced neuronal apoptosis (Fig. 6a). Axonal degeneration in neurons is a common neuropathological change that can be caused by factors such as metabolic disturbances and immune-mediated inflammation [43]. Immunofluorescence results indicated that conditioned media from *ST2* knockdown BV2 cells induced HT22 neuronal apoptosis, and caused axonal degeneration from the distal to the proximal end, with damage and disappearance of the axon and loss of axonal nodes. In contrast, HT22 neurons treated with IL-33 show better axonal morphology and reduced axonal breakage (Fig. 6b). These findings suggest that IL-33/ST2 signaling in BV2 cells is essential for their neuroprotective effects in vitro.

### Exogenous IL-33 improved the cognitive outcome of rmTBI mice

**Fig. 7:**
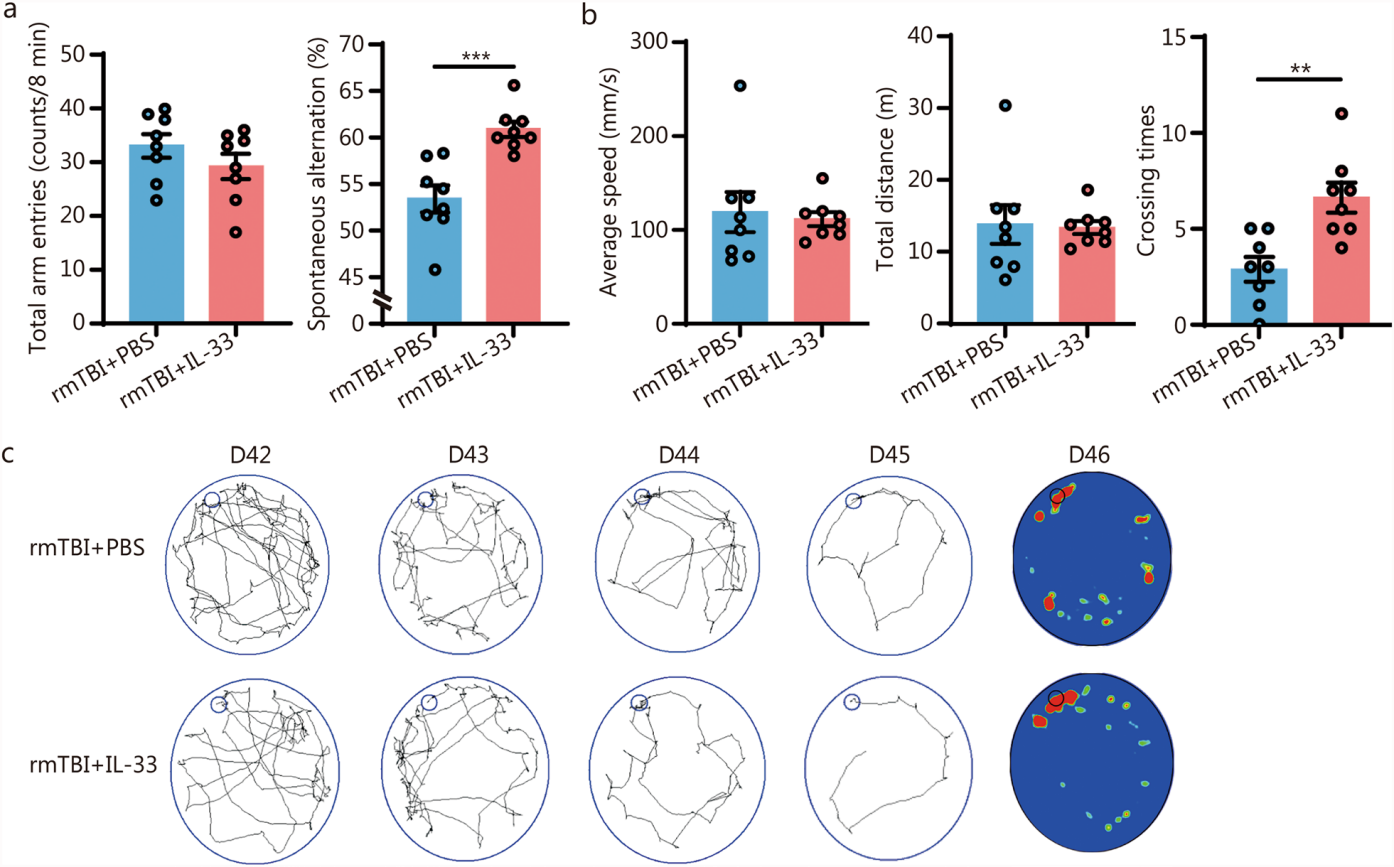
Exogenous IL-33 improved the cognitive outcome of rmTBI mice. **a** Total number of entries and percentage of spontaneous alternations in the Y-maze (*n* = 8). **b** Average speed, total distance, and crossing times during the exploration phase of the Barnes maze for mice (*n* = 8). **c** Training and spatial exploration paths of mice in the Barnes maze experiment from days 42 to 46 post-rmTBI. Data are represented as the mean ± SEM. ^**^P < 0.01, ^***^P < 0.001. rmTBI repetitive mild traumatic brain injury, PBS phosphate buffer saline, IL-33 interleukin-33

Intranasal administration is a non-invasive mode of drug delivery, in which the drug is delivered directly to the systemic or CNS through the nasal mucosa. This approach offers advantages such as rapid absorption, avoidance of first-pass effects, and targeted delivery to the CNS. It has been used for treating conditions, such as AD, PD, and depression [24]. We administered exogenous recombinant IL-33 protein (2 µg per 30 g of body weight) intranasally to mice on days 0, 7, 14, 21, 28, 35, and 42 following rmTBI. On 42 d post-injury, we evaluated the memory and learning abilities of the mice using the Y-maze and Barnes maze tests. We observed that the spatial memory learning capabilities of rmTBI mice were impaired vs. the sham mice. However, upon the supplementation of IL-33, there was a marked increase in the number of mice successfully navigating to the target hole in the Barnes maze. Similar results were obtained from the Y-maze test. These findings indicated that exogenous IL-33 significantly enhanced spatial memory and alleviated cognitive function deficits induced by rmTBI (Fig. 7a-c).

## Discussion

**Fig. 8:**
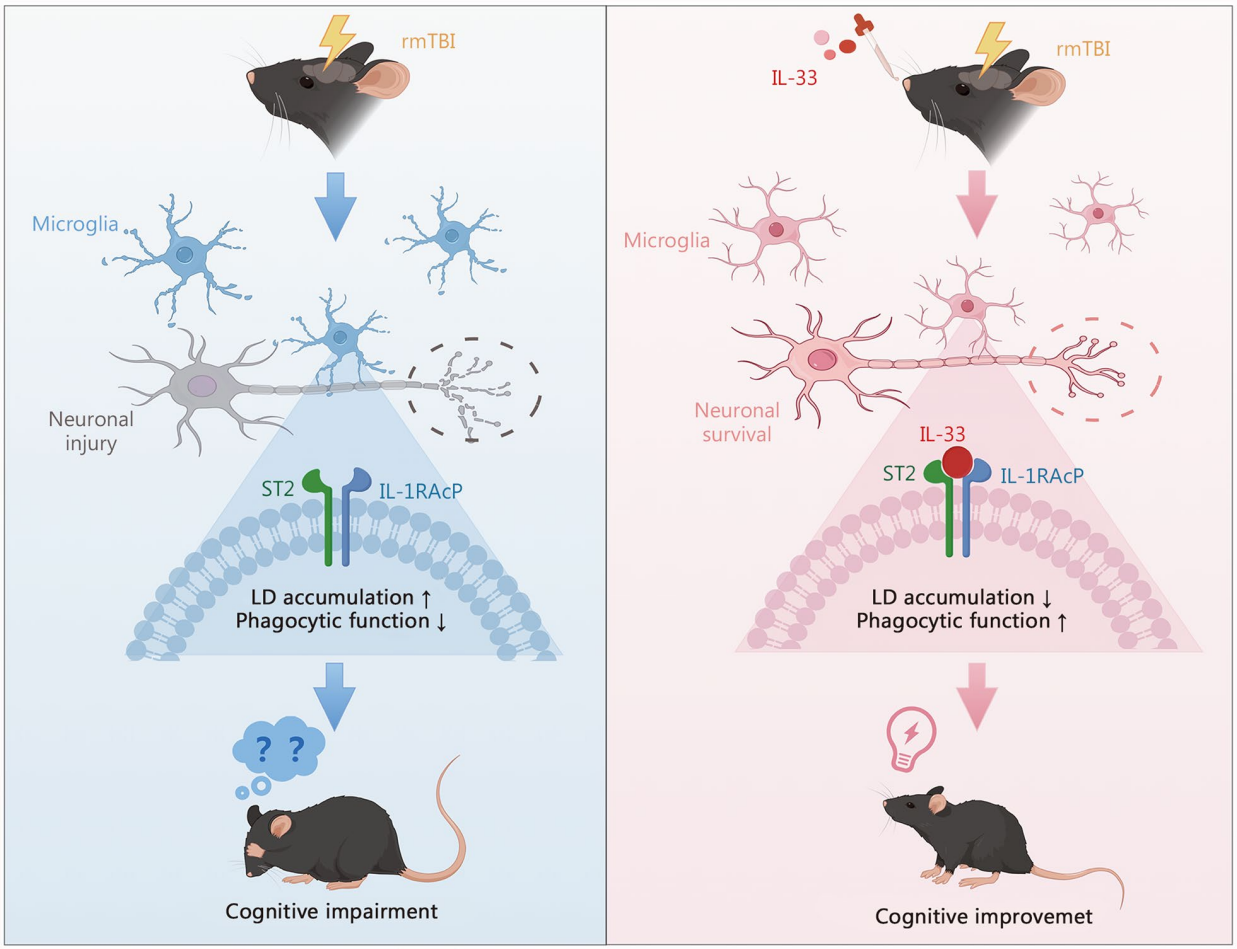
Intranasal administration of the recombinant IL-33 protein attenuated the accumulation of microglial lipid droplets, enhanced Aβ phagocytosis, and promoted neuronal survival in the brain, thus contributing to improving the cognitive outcome of rmTBI mice. rmTBI repetitive mild traumatic brain injury, IL-33 interleukin-33, ST2 suppression of tumorigenicity 2, IL-1RAcP interleukin-1 receptor accessory protein, LD lipid droplet, Aβ amyloid-beta

As the incidence of chronic neurodegenerative diseases resulting from rmTBI increases, the condition has attracted significant attention, yet its pathogenesis remains largely unclear. In this study, various methods were adopted to demonstrate that IL-33 deficiency exacerbated neurocognitive dysfunction following rmTBI. Elevated IL-33 levels were detected in the blood and extracellular vesicles of rmTBI patients relative to healthy controls. A similar phenomenon was observed in the rmTBI mouse model. Specifically, the expression level of IL-33 in the brain tissue was elevated at day 3 post-injury, but decreased by day 42. To further explore IL-33 expression in the CNS, immunofluorescence was used, and found that astrocytes primarily produced IL-33, while its receptor ST2 was mainly expressed in microglia. Behavioral tests, including the Y-maze and Barnes maze, showed that IL-33 deficiency worsened spatial learning and memory deficits after rmTBI. Besides, PET/CT imaging revealed that IL-33 deficiency promoted the expression of neurodegenerative proteins, such as APP and tau, as well as elevated pro-inflammatory factors in the brain. To further investigate how IL-33 manipulated the development of neurodegeneration, transcriptomic sequencing was conducted, which demonstrated an enrichment of phagocytosis in rmTBI mice. Given that the ST2 receptor is predominantly expressed in microglia, we then focused on microglia and found that IL-33 deficiency reduced microglial phagocytic activity and impaired their ability to clear pathological proteins, thereby exacerbating cognitive impairment after rmTBI. Previous studies have reported that in aging and neurodegenerative diseases such as AD, microglia exhibit abnormal lipid accumulation of LDs [44, 45]. LPS-stimulated BV2 cells exhibited impaired phagocytic activity upon excessive LD accumulation, which was restored by IL-33 treatment. Additionally, IL-33/ST2 signaling activation was crucial for the neuroprotective properties of BV2 cells in vitro. Consequently, the IL-33 supplementation therapy was designed and found to be effective in improving neurodegeneration and cognitive outcome of rmTBI mice (Fig. 8).

The relationship between peripheral and central IL-33 expression following rmTBI remains unclear. Peripheral IL-33 was upregulated in both rmTBI patients and experimental rmTBI mice, with concurrently elevated IL-33 levels detected in peripheral exosomes. This leads us to hypothesize that peripheral IL-33 may increase compensatorily to replenish central IL-33 levels. Peripheral endothelial cells released IL-33-containing exosomes under inflammatory conditions. This finding suggests that endothelial cells may play an important role in the development of inflammation, but whether the exosomes enter the CNS or peripheral immune organs (e.g., spleen) remains unclear. Future studies should investigate the in vivo distribution and function of IL-33-containing exosomes to elucidate their potential regulatory roles in neuroinflammatory responses. To validate this hypothesis, future experiments could specifically inhibit the secretion of endothelial cell exosomes, such as using GW4869 [46], to investigate the precise origin of central IL-33 following rmTBI. Concurrent with in vitro microglia-neuron interaction studies, cytokine profiling of microglial conditioned medium can be performed.

IL-33 has a wide range of roles in a variety of diseases and homeostatic conditions. (1) In physiological conditions, IL-33 plays a key role in maintaining tissue homeostasis as an alarmin in epithelial cells and barrier tissues. When tissues are damaged or infected, IL-33 is released from damaged cells and activates immune cells to help the body resist pathogen infections and repair tissue damage [19, 47]. (2) IL-33 is a multifunctional cytokine that not only plays a role in the immune response, but also in multiple physiological and pathological processes such as tissue repair and inflammation regulation [28, 33]. (3) IL-33 is aberrantly expressed in a variety of diseases, such as asthma, allergic reactions, cardiovascular diseases, and autoimmune diseases. IL-33 regulates the balance between pro- and anti-inflammatory cytokines. It also releases neurotrophic factors, which have a protective effect on nerve cells in the CNS [13, 35]. There are 2 points for the clinical assessment of IL-33 in rmTBI. (1) Auxiliary evaluation tools: despite the limited specificity of IL-33 as a single marker, its use in combination with other biomarkers may be considered a reasonable strategy to improve diagnostic accuracy and specificity. (2) Individualized assessment: considering that changes in IL-33 may vary due to individual differences, establishing personalized baseline levels of IL-33 and monitoring its dynamic changes at different time points post-injury may be an effective strategy to improve its diagnostic value as a druggable marker.

Growing evidence indicates that IL-33 plays a significant role in the development of CNS disorders. In the APP/presenilin 1 mouse model of AD, IL-33 enhances the chemotactic response of microglia, promoting their movement toward Aβ plaques and aiding in Aβ clearance. This mechanism contributes to a reduction in AD-related pathology and improvements in memory impairments [48]. In experimental autoimmune encephalomyelitis, IL-33 treatment reduces the concentrations of IL-17 and IFN-γ, alleviating disease progression [49]. Furthermore, the absence of ST2 augments the differentiation of pro-inflammatory antigen-presenting cells and inflammatory T cells, thereby diminishing the mice’s resilience to experimental autoimmune encephalomyelitis [50]. Prolonged IL-33 administration delays the onset of the disease in transgenic mice afflicted with amyotrophic lateral sclerosis and attenuates astrocyte activation [51]. Several monoclonal antibodies targeting IL-33 have been developed, primarily for conditions such as chronic obstructive pulmonary disease and asthma. For example, Tozorakimab, a monoclonal antibody targeting IL-33, effectively inhibits IL-33-driven, ST2-dependent inflammatory responses. Results from the phase I clinical trials showed that Tozorakimab significantly reduced serum levels of pro-inflammatory cytokines, particularly IL-5 and IL-13, in patients with chronic obstructive pulmonary disease [52]. Although there is currently no research on IL-33-targeted therapies in the field of TBI, the above studies illustrate the central role of IL-33 in the inflammatory response. IL-33 as a promising target may provide new strategies for the treatment of TBI and neurodegenerative diseases in the future.

As the most important polymorphic lipid transport protein in the human body, apolipoprotein E (ApoE) plays a central role in the regulation of lipid metabolism. Its main functions include mediating the transport and metabolic balance of triglyceride and cholesterol: on the one hand, it promotes the utilization and storage of lipids in peripheral tissues, and on the other hand, it dynamically regulates lipid clearance and anabolic metabolism according to the needs of the body [53]. There are 3 major allelic variants of the ApoE gene (*ApoE2*, *ApoE3*, and *ApoE4*), of which *ApoE4* is recognised as an important genetic risk factor for AD [54, 55]. In recent years, a subpopulation of microglia with a specific activation state has been found in the brain tissue of AD patients, characterised by high expression of acyl-CoA synthetase long-chain family member. Notably, these acyl-CoA synthetase long-chain family member 1^+^ microglia showed a specific enrichment in the brains of AD patients with the ApoE4 pure-blood genotype (ApoE4/4) [41]. In neuroinflammatory contexts, ApoE (particularly the E4 isoform) modulates microglia/macrophage activation states, thereby influencing inflammatory factor release. *ApoE* deficiency or dysfunction may lead to an enhanced pro-inflammatory environment, which in turn upregulates APP expression. In terms of AD pathogenesis, ApoE4 may contribute to disease progression through several pathways. (1) Regulating APP metabolism: significantly increasing Aβ production by enhancing the recycling process of APP and upregulating the expression level of β-secretase [56]. (2) Affecting Aβ clearance: not only decreasing the cellular uptake of Aβ, but also inhibiting the activity of Aβ-degrading enzymes [57]. (3) Mediating neuroinflammatory responses: in the neuroinflammatory microenvironment, Aβ induces an ApoE-dependent reprogramming of lipid metabolism in microglia, as manifested by increased triglyceride synthesis, abnormal accumulation of LDs, and release of neurotoxicity factors. These pathological changes may further lead to neuronal degeneration through lipid transfer [41, 58].

As the primary defenders of the CNS, microglia play a crucial role in synaptic pruning, injury repair, and maintenance of homeostasis [13, 59–61]. In pathological conditions, microglia primarily function by phagocytosing tissue debris and secreting various cytokines, growth factors, and chemokines [32]. In AD, impaired microglial phagocytosis leads to the accumulation of Aβ and the formation of amyloid plaques [17]. Therefore, it is essential to clarify the role of microglia in rmTBI. In addition to microglia, the ST2 receptor is also highly expressed in astrocytes within the CNS, suggesting that astrocytes may also respond to IL-33/ST2 signaling. Previous studies have shown that IL-33 can reduce astrocyte activation in the ischemic penumbra following stroke [62–64]. Further research is essential to clarify the role and effects of IL-33 on astrocytes in the context of rmTBI. A deeper understanding of how IL-33 influences astrocyte responses could provide valuable insights into the mechanisms of rmTBI and guide the development of therapeutic strategies to mitigate its effects.

Despite efforts to ensure the comprehensiveness of our study, some limitations remain that are worth further exploration in future research. (1) Clinical sample limitations. As a rigorously controlled pilot study, recruiting a large number of patients with rmTBI was challenging. Specifically, most of the patients included in this study were elderly individuals with multiple fall injuries due to difficulties in mobility and active/retired full-contact sports athletes (boxing, judo, wrestling, etc.). In contrast, the vast majority of patients with rmTBI do not go to the hospital due to a lack of awareness of the disease. This has resulted in an extremely low diagnosis rate. However, this part of the work was focused on making a preliminary assessment of IL-33 expression in the blood of rmTBI patients. In the future, we plan to include more subjects through multicenter collaboration to further validate and explore the present findings. (2) Animal models. The CCI device delivers mechanical impacts at a fixed trajectory, which fails to replicate the complex rotational acceleration forces characteristic of clinical head injuries. However, the CCI model has its irreplaceable advantages in that the damage parameters of the instrument are precisely controllable (velocity, depth, residence time), which makes it suitable for mechanism studies [25, 26, 39, 65]. (3) Cell line selection. The BV2 and HT22 cell lines used in our in vitro experiments may not fully represent primary cell functions in vivo. Limitations include: BV2 cells are immortalized and may differ in phenotype and function from primary microglia, lacking full in vivo behaviors like phagocytosis and intercellular communication. HT22 neurons may lack key features such as synaptic plasticity. Our in vitro conditions using LPS to activate Toll-like receptor 4 (TLR4) in BV2 cells simplify the complex environment of rmTBI, which typically involves multiple receptor pathways like TLR2, TLR4, and the receptor for advanced glycation end products. (4) Potential effects on human patients. Although IL-33, the subject of this study, was found in a human study, the extrapolation of the results should be cautious because the main conclusions are derived from the results of animal experiments and cell line studies. Future research should use primary microglia and neurons to further validate the role of IL-33 in response to cell injury and assess its potential impact on human patients.

## Conclusions

In conclusion, our findings suggest that IL-33 exerts neuroprotective effects by regulating microglial phagocytosis through the IL-33/ST2 axis. These findings suggest that IL-33-mediated signaling plays a critical role in alleviating neurodegeneration after rmTBI, highlighting its potential as a therapeutic target for cognitive recovery in patients.

## Supplementary Information

**Additional file 1**. Methods. **Table S1** Baseline characteristics of 6 paired patients with rmTBI and control individuals. **Fig. S1** Cellular localization of ST2 in the hippocampal (dentate gyrus) and cortical regions of WT-rmTBI mice. **Fig. S2** The expression of *Fcgr4* was decreased in IL-33KO group mice after rmTBI. **Fig. S3** Morphology and function of microglia.

## Data Availability

Source data are included in this original research article. Any additional data requests are available from the corresponding author upon request.

## Abbreviations

AD: Alzheimer’s disease
Aβ: Amyloid-beta
ApoE: Apolipoprotein E
APP: Amyloid precursor protein
CCI: Controlled cortical impact
CNS: Central nervous system
CT: Computed tomography
CTE: Chronic traumatic encephalopathy
DMEM: Dulbecco’s modified Eagle medium
FITC: Fluorescein isothiocyanate
GFAP: Glial fibrillary acidic protein
GSEA: Gene Set Enrichment Analysis
Iba1: Ionized calcium-binding adapter molecule 1
IL-33: Interleukin-33
KEGG: Kyoto Encyclopedia of Genes and Genomes
LAMP1: Lysosome-associated membrane protein 1
LDs: Lipid droplets
LPS: Lipopolysaccharide
NeuN: Neuron-specific nuclear protein
NTA: Nanoparticle tracking analysis
Olig2: Oligodendrocyte lineage transcription factor 2
PBS: Phosphate buffer solution
PD: Parkinson’s disease
PET: Positron emission tomography
rmTBI: Repetitive mild traumatic brain injury
RT-qPCR: Real-time quantitative polymerase chain reaction
ST2: Suppression of tumorigenicity 2
TBI: Traumatic brain injury
TEM: Transmission electron microscope
TGF-β: Transforming growth factor-β
TLR4: Toll-like receptor 4
TMEM119: Transmembrane protein 119
TNF-α: Tumor necrosis factor-α
vWF: Von Willebrand factor
WT: Wild-type
