# Supplementary material for: Decreased IL-33 in the brain following repetitive mild traumatic brain injury contributes to cognitive impairment by inhibiting microglial phagocytosis
Source: Mil Med Res. 2025 Aug 5;12:46. doi: 10.1186/s40779-025-00631-1 (PMC12323175; doi:10.1186/s40779-025-00631-1)
Supplement: Supplementary file 1 — Additional file 1. Methods. Table S1 Baseline characteristics of 6 paired patients with rmTBI and control individuals. Fig. S1 Cellular localization of ST2 in the hippocampal (dentate gyrus) and cortical regions of WT-rmTBI mice. Fig. S2 The expression of Fcgr4 was decreased in IL-33KO group mice after rmTBI. Fig. S3 Morphology and function of microglia. [file 40779_2025_631_MOESM1_ESM.pdf]

Methods

Barnes maze test

The Barnes maze test was performed on day 42 post-rmTBI [1]. A single acclimation session was performed before 4 consecutive days of testing. During the test, mice instinctively seek a dark, enclosed space, represented by an escape route located beneath the target hole. On the 5th day, the escape route was removed, and an exploration trial was conducted in which the mice freely explored for 2 min. All procedures were recorded and analyzed blindly. Spatial learning and memory capabilities were assessed by counting the number of crossings to the target hole.

Y-maze test

The Y-maze test was used to evaluate the spatial working memory of the animals through spontaneous alternation [2]. The Y-maze apparatus consisted of a central area and 3 arms of equal size (30 cm × 8 cm × 15 cm), labeled A, B, and C. Mice were allowed to explore these arms freely from the central area for 8 min, and the number of complete limb entries into each arm was recorded.

Extraction of exosomes

Extracellular vesicles were isolated using the Total Exosome Isolation Kit (Invitrogen, USA) [3]. Initially, samples were centrifuged at 10,000 × *g* for 20 min. The clarified supernatant was carefully aspirated, and 0.5 volumes of phosphate buffer solution (PBS) along with 0.2 volumes of exosome precipitation reagent were added. The mixture was incubated for 10 min. Finally, centrifugation at 10,000 × *g* for 5 min was performed, and the supernatant was discarded. The precipitate was resuspended in PBS to isolate the exosomes.

Characterization of purified exosomes

Micrographs were observed using a transmission electron microscope (TEM) (Hitachi, Japan).

Nanoparticle tracking analysis (NTA) was used to assess the size distribution of exosomes extracted from mouse or patient blood samples.

Real-time quantitative polymerase chain reaction (RT-qPCR)

Total RNA was extracted from different samples using TRIzol reagent (Invitrogen, USA). cDNA was synthesized using the Reverse Transcription Kit (TransGen Biotech, China) according to the instructions provided by the manufacturer. RT-qPCR was performed using SYBR Green Master Mix (TransGen Biotech) and detected on a real-time PCR detection system. Fold change was calculated using the formula $2^{-\Delta\Delta CT}$, where ΔCT = CT value of the target gene – CT value of the internal reference gene, and $\Delta\Delta CT$ = ΔCT value of the experimental group – ΔCT value of the control group. We used the internal reference gene (*β-actin*) to correct for differences between samples to ensure the accuracy of the experimental results.

Mouse enzyme-linked immunosorbent assay (ELISA) kit

Mouse brain tissues were homogenized with saline, centrifuged, and the supernatants were collected. Samples, standards, and detection antibodies were added to microtiter plates. After incubation, a substrate was introduced to facilitate color development, and the optical density was measured at 450 nm.

Sholl analysis of microglial morphology

$Iba1^{+}$ microglia were imaged using confocal microscopy (Z-stacks). Only microglia with complete, non-overlapping processes were selected for analysis. The soma centroid was manually defined as the center point for Sholl analysis. The maximum process length (distance from the soma centroid to the terminal point of the longest process) was measured using Fiji's line tool. Intersections between microglial processes and each concentric circle were automatically quantified using Fiji's Sholl

analysis plugin.

Immunofluorescence and BODIPY staining

Cells were seeded on poly-L-lysine-coated coverslips, fixed with 4% paraformaldehyde for 20 min, and washed each well 3 times in PBS. Blocking was performed for 1.5 h. Primary antibodies were incubated overnight at 4 °C. The primary antibodies used are: ionized calcium-binding adapter molecule 1 (Iba1, 1:200, OB-PGP049, Oasis Biofarm, China) and β III tubulin (1:200, ab78078, Abcam, UK). For BODIPY staining, BODIPY 493/503 dye (1:500, GlpBio, USA) was mixed with secondary antibodies and incubated at 37 °C for 1 h.

In vitro amyloid-beta (Aβ) phagocytosis assay

Following established protocols, fluorescein isothiocyanate (FITC)-Aβ1-42 (Anaspec, USA) was aggregated at 37 °C for 24 h with agitation. BV2 cells were plated in black-walled 96-well plates and cultured overnight. Before fluorescence measurement, the Aβ-containing medium was removed, and the cells were washed twice with pre-warmed Dulbecco's modified Eagle medium (DMEM) to eliminate extracellular Aβ. Fluorescence was measured at 485 nm excitation and 538 nm emission [4].

Apoptosis assay

HT22 cell apoptosis was detected by flow cytometry. Cells were digested with trypsin (EDTA-free) and washed once with PBS. After resuspension, Annexin V-APC and 7-AAD Percp-CY5.5 were added, and the cells were incubated in the dark for 15 min. After filtering, cells were analyzed by flow cytometry (BD, USA) using FlowJo 10.8. The gating strategy used in the flow cytometry analysis of HT22 neuronal cell apoptosis was as follows. (1) Debris and cell aggregates were excluded, and live cell populations with high FSC-A (representing cell size) and medium SSC-A (representing granularity) were retained. (2) Duplex or multiplex cells were based on the linear relationship between the height

(FSC-H) and area (FSC-A) of the forward scatter, ensuring that the analysis was based on single cells. (3) In single cell populations, results were quantified by the Annexin V^+/7-AAD^+ ratio according to the fluorescence signals of Annexin V-APC and 7-AAD Percp-CY5.5 [5-8].

References

1. Zuo W, Zhao J, Zhang J, Fang Z, Deng J, Fan Z, et al. MD2 contributes to the pathogenesis of perioperative neurocognitive disorder via the regulation of $\alpha 5GABA_A$ receptors in aged mice. J Neuroinflammation. 2021;18(1):204.
2. Kraeuter AK, Guest PC, and Sarnyai Z. The Y-maze for assessment of spatial working and reference memory in mice. Methods Mol Biol. 2019;1916:105-11.
3. Chen G, Huang AC, Zhang W, Zhang G, Wu M, Xu W, et al. Exosomal PD-L1 contributes to immunosuppression and is associated with anti-PD-1 response. Nature. 2018; 560(7718): 382-6.
4. Xiang X, Werner G, Bohrmann B, Liesz A, Mazaheri F, Capell A, et al. TREM2 deficiency reduces the efficacy of immunotherapeutic amyloid clearance. EMBO Mol Med. 2016;8(9):992-1004.
5. Bi W, Li X, Jiang Y, Gao T, Zhao H, Han Q, et al. Tumor-derived exosomes induce neutrophil infiltration and reprogramming to promote T-cell exhaustion in hepatocellular carcinoma. Theranostics. 2025;15(7):2852-69.
6. Vijeyakumaran M, Jawhri MA, Fortunato J, Solomon L, Shrestha Palikhe N, Vliagoftis H, et al. Dual activation of estrogen receptor alpha and glucocorticoid receptor upregulate CRTh2-mediated type 2 inflammation; mechanism driving asthma severity in women? Allergy. 2022;78(3):767-79.
7. Oudaert I, Satilmis H, Vlummens P, De Brouwer W, Maes A, Hose D, et al. Pyrroline-5-carboxylate reductase 1: a novel target for sensitizing multiple myeloma cells to bortezomib by inhibition of PRAS40-mediated protein synthesis. J Exp Clin Cancer Res. 2022;41(1):45.
8. Cai Y, Chen X, Lu T, Fang X, Ding M, Yu Z, et al. Activation of STING by SAMHD1 deficiency promotes PANoptosis and enhances efficacy of PD-L1 blockade in diffuse large B-cell lymphoma. Int J Biol Sci. 2023;19(14):4627-43.

Table S1 Baseline characteristics of 6 paired patients with rmTBI and control individuals

Subjects number	Age	Gender	mTBI degree	Time since last injury	MoCA score	MMSE score	RPQ-3 score	RPQ-16 score	History
rmTBI #1	41	Female	FCS 15 years	4 years	28	30	1	2	Forgetfulness (1); Irritable (1)
Control #1	41	Female	None	-	30	30	0	0	-
rmTBI #2	41	Female	FCS 13 years	4 years	24	26	5	11	Headache (1); Dizziness (1); Forgetfulness (3); Nausea (1); Fatigue (1); Depressed (1); Poor concentration (1); Longer to think (1); Restless (1)
Control #2	40	Female	None	-	30	30	0	0	-
rmTBI #3	44	Female	FCS 10 years	1 year	22	25	3	19	Dizziness (1); Forgetfulness (2); Sleep disturbance (4); Fatigue (2); Irritable (3); Depressed (2); Frustrated (2); Poor concentration (1); Longer to think (1); Blurred vision (1)
Control #3	43	Female	None	-	30	30	0	0	-
rmTBI #4	62	Male	4 times	1 year	20	22	4	16	Headache (1); Dizziness (2); Forgetfulness (1); Nausea (1); Sleep disorder (4); Fatigue (1); Irritable (1) Depressed (1); Frustrated (1); Poor concentration (1); Longer to think (1); Restlessness (1)
Control #4	59	Male	None	-	28	30	0	3	Sleep disorder (1); Fatigue (1); Irritable (1)
rmTBI #5	62	Male	2 times	1 year	28	30	0	3	Fatigue (1); Irritable (1); Frustrated (1)
Control #5	58	Male	None	-	29	30	0	2	Fatigue (2)
rmTBI #6	71	Female	4 times	3 years	22	24	3	12	Headache (1); Forgetfulness (2); Sleep disturbance (2); Fatigue (1); Irritable (2); Poor concentration (2); Longer to think (2)

Subjects number	Age	Gender	mTBI degree	Time since last injury	MoCA score	MMSE score	RPQ-3 score	RPQ-16 score	History
Control #6	68	Female	None	-	26	28	1	5	Forgetfulness (1); Sleep disturbance (1); Fatigue (1); Irritable (1); Longer to think (1)

Degree of symptoms: (1) no more of a problem, (2) a mild problem, (3) a moderate problem, (4) a severe problem. *FCS* full contact sports, *MMSE* mini-mental state examination, *MoCA* montreal cognitive assessment, *RPQ* rivermead post-concussion symptoms questionnaire, *rmTBI* repetitive mild traumatic brain injury

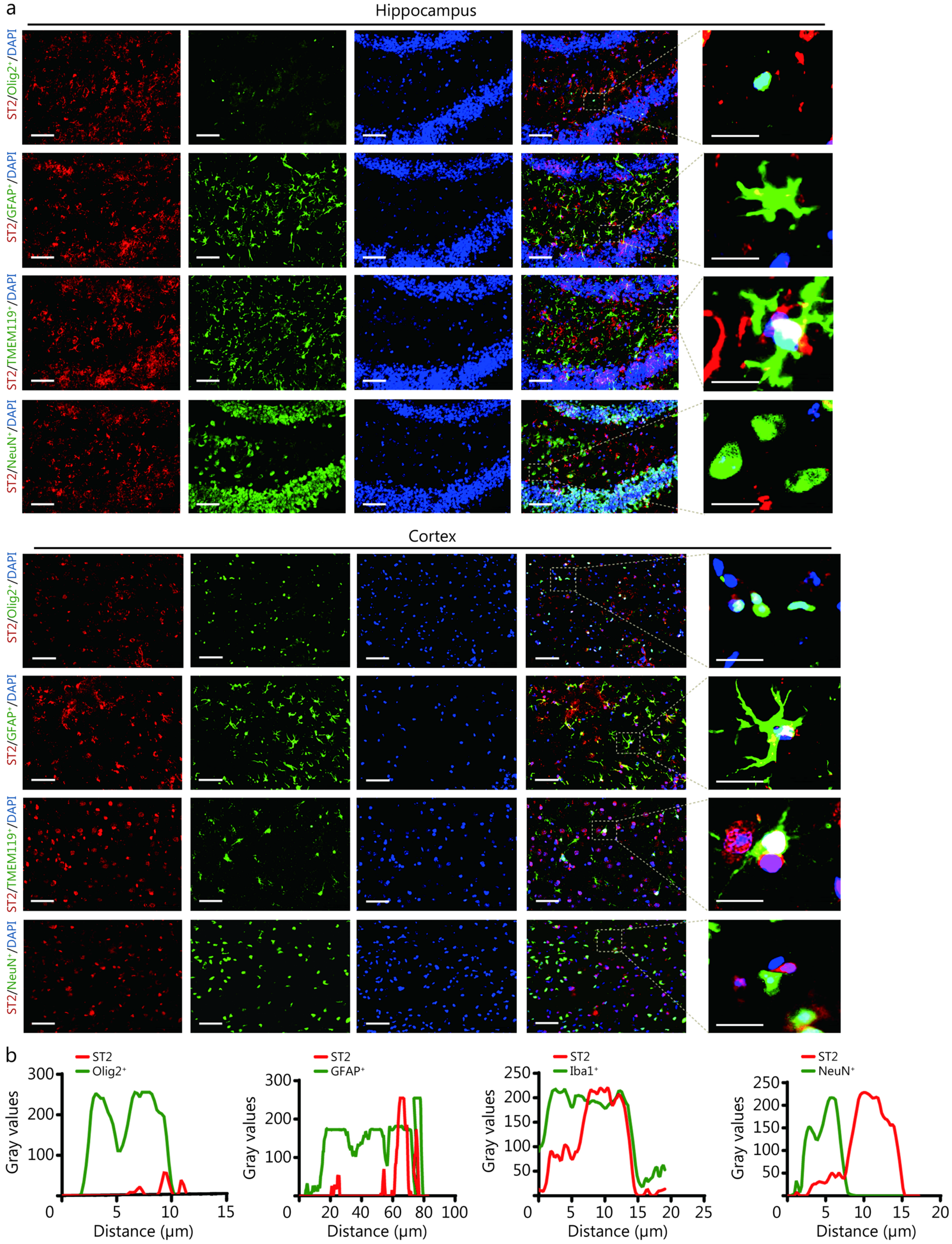

Fig. S1 Cellular localization of ST2 in the hippocampal (dentate gyrus) and cortical regions of WT-rmTBI mice. **a** Representative immunofluorescence staining images of ST2 (red), Olig2^{+} oligodendrocytes (green), GFAP^{+} astrocytes (green), Iba1^{+} microglia (green), NeuN^{+} neurons, and DAPI (blue) in the hippocampus and cortex of mice on day 42 post-rmTBI (*n* = 5, 3 slides/mouse. Consistent fields of view were selected across all slides for subsequent quantification). Scale bar = 50

μm (main images) and 25 μm (magnified insets). **b** Fluorescence intensity plots of ST2 and various cells. The red curves show the relative intensity of ST2, and the green curves show Olig2^{+} oligodendrocytes, GFAP^{+} astrocytes, Iba1^{+} microglia, and NeuN^{+} neurons. DAPI 4,6-diamidino-2-phenylindol dihydrochloride, ST2 suppression of tumorigenicity 2, Olig2 oligodendrocyte lineage transcription factor 2, GFAP glial fibrillary acidic protein, Iba1 ionized calcium-binding adapter molecule 1, NeuN neuron-specific nuclear protein, WT wild-type, rmTBI repetitive mild traumatic brain injury

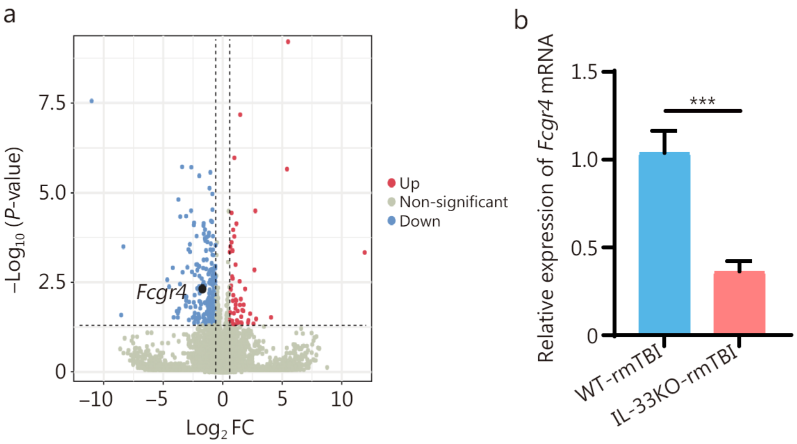

Fig. S2 The expression of *Fcgr4* was decreased in IL-33KO group mice after rmTBI. **a** Volcano plot of up- and down-regulated genes. Red dots indicate up-regulated genes in the IL-33KO-rmTBI group, and blue dots indicate down-regulated genes. The significantly different genes were identified using $P < 0.05$ and fold change (FC) > 2 or < 0.5. **b** Relative mRNA expression of *Fcgr4* in the brains of mice in the WT-rmTBI and IL-33KO-rmTBI groups was detected by RT-qPCR ($n = 6$). Data are expressed as mean ± SEM. $^{***}P < 0.001$. IL-33 interleukin-33, rmTBI repetitive mild traumatic brain injury, WT wild-type, KO knockout, RT-qPCR real-time quantitative polymerase chain reaction

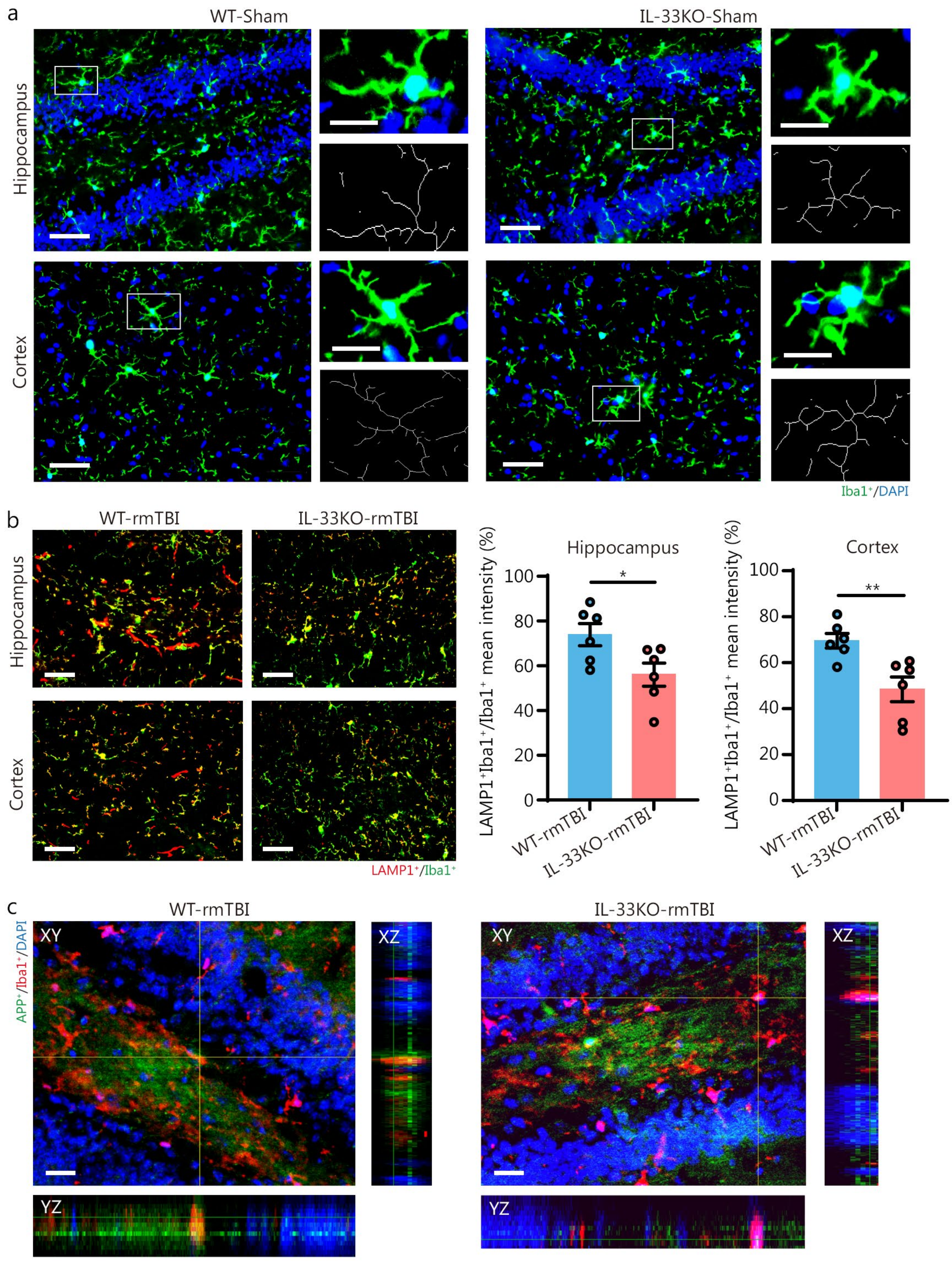

Fig. S3 Morphology and function of microglia. **a** Representative immunofluorescence staining image of Iba1^{+} microglia (green) and DAPI (blue) in the hippocampus and cortex of WT-Sham and IL-33KO-Sham mice. Scale bar = 50 μm (main images) and 20 μm (magnified insets). **b** Representative immunofluorescence staining images and quantification of LAMP1^{+} (red), Iba1^{+} microglia (green) in the hippocampus and cortex of WT and IL-33KO mice after rmTBI (*n* = 6). Scale bar = 50 μm. **c** Orthogonal views of representative immunofluorescence staining for APP^{+} (green), Iba1^{+} microglia (red), and DAPI (blue) in the hippocampus of WT mice and IL-33KO group mice after rmTBI. Scale

bar = 20 μm. Data are represented as the mean ± SEM. $^{*}P < 0.05$, $^{**}P < 0.01$. DAPI 4,6-diamidino-2-phenylindole dihydrochloride, IL-33 interleukin-33, LAMP1 lysosome-associated membrane protein 1, rmTBI repetitive mild traumatic brain injury, APP amyloid precursor protein, Iba1 ionized calcium-binding adapter molecule 1, WT wild-type, KO knockout
